# Adding a Seat at the Table: A Case Study of the Provider's Perspective on Integrating Community Health Workers at Provider Practices in California

**DOI:** 10.3389/fpubh.2021.690067

**Published:** 2021-10-28

**Authors:** Courtney A. Paulson, Eva M. Durazo, Leigh D. Purry, Arianne E. Covington, Bruce Alan Bob, Rebecca A. Peters, Steven Torchia, Baylis Beard, Lucy E. McDermott, Amy Lerner, Joycelyn Smart-Sanchez, Mahima Ashok, Jacqueline Ejuwa, Shannon Cosgrove

**Affiliations:** ^1^Blue Shield of California, Health Transformation and Network Management, Oakland, CA, United States; ^2^Hill Physicians Medical Group, Sacramento, CA, United States; ^3^Capital OBGYN, Hill Physicians Medical Group, Sacramento, CA, United States

**Keywords:** community health worker (CHW), health equity, social determinants of health (SDOH), provider integration, community health, social needs, holistic health

## Abstract

Blue Shield of California's Community Health Advocate Program was created to support whole person-health needs by helping individuals of all socio-economic statuses navigate and access community resources, social services, and medical systems. Blue Shield's Health Reimagined team is partnering with medical providers, community resources centers, and community partners to provide intensive person-centered and technology-enabled care to patients, ensuring social needs are met while promoting health equity. A key aspect of the Health Reimagined initiative embeds Community Health Advocates (CHAs) within physician practices serving patients using a payor-agnostic approach, by which Blue Shield aims to increase access to social services and community resources, improve health outcomes, reduce medical costs, and improve overall patient experience. The purpose of this case study is to understand the provider's perspective of embedding a CHA into the care team and the resulting impact on the practice and patients. Blue Shield also sought to identify best practices and barriers of a CHA program within primary and specialty care practices. As part of an ongoing two-year mixed-methods impact evaluation (2019–2021), 10 semi-structured interviews were conducted with a total of 18 providers and office staff at five primary care and specialty practices where CHAs have been embedded. We also conducted two focus groups with the same five CHAs at different points in time. Several themes emerged from the provider, office staff, and CHA interviews. Provider practices found great value in adding a CHA to their care team as the CHA brings flexibility and continuity to patient care. They also found that having access to a CHA with shared life experiences of the communities they served is a key component to the program's success. Providers and staff reported a new understanding of the social determinants of health that impacts a patient's wellbeing with the embedding of a CHA in the care team. Overall, practitioners expressed high satisfaction with the CHA program. During the COVID-19 pandemic, CHAs have been critically important in care, as social needs have increased, and resources have shifted. The CHA program is constantly adapting to address challenges faced by all stakeholders and applying new knowledge to ensure best practices are implemented within the CHA program.

## Introduction

With the various health needs of diverse populations and the limited resources available to support these needs, it is imperative to support advocacy initiatives that transform community health (1). Social Determinants of Health, or the inherent conditions in which people live, learn, work, and play, often affect a wide range of health risks and outcomes.^1^ Research has shown that up to 80% of a patient's health is impacted by social determinants (2), and these needs may be better addressed outside of traditional health care delivery systems. In 2020, the widespread impacts of the COVID-19 pandemic on historically marginalized communities have made the need for holistic, community-integrated care even more urgent and visible.

Community Health Workers/Promotores (CHW/Ps) are positioned to provide support and identify patients' social needs, help navigate the medical system, and provide referrals and connections to community resources. These public health workers often share a sociocultural background with the patients in the communities they serve, which allows them to establish trust and improve the health system's ability to provide higher quality and culturally appropriate care (3). Evidence shows that CHW/P interventions can improve health outcomes for marginalized communities by increasing access to primary care, improving behavioral health, reducing the likelihood of 30-day hospital readmissions, supporting chronic disease management, and lowering hospitalization rates (4–7).

CHW/Ps are traditionally managed by community-based organizations or organizations that function as the liaison between health systems and communities (8–10). With increased financing and delivery of health care services, a recent approach to CHW/Ps as an extension of the clinical system has been introduced that blends these traditional approaches and expands the CHW/P role into healthcare settings. As members of the clinical care team, CHW/Ps can help improve health care systems to be more appropriate and accessible for community members by shifting to a patient-centered, preventative approach to care (11). Integrating CHW/Ps into multidisciplinary care settings allows CHW/Ps to take on a wider spectrum of responsibilities and complement the skills of clinical staff to support complex medical and non-medical care needs that go beyond the walls of the clinic (12).

While research has shown that integrating CHW/Ps into primary care settings is effective and the interventions can result in improved health outcomes,^2^ evidence is still developing on how to assess and improve organizational readiness for integration into primary and specialty care settings. The healthcare system lacks a widespread care delivery system and readiness effort aimed at integrating the CHW/P role into coordinated care delivery models. Understanding and respecting the CHW/P model within the clinical setting is essential for CHW/Ps to be fully integrated within care delivery systems and utilized to the fullest potential (9).

The purpose of this case study is to identify best practices and barriers of a CHW/P program established by Blue Shield of California (Blue Shield). The study shares background about the CHW/P program's approach and findings to date related to the recruitment and training, but primarily the integration of CHW/Ps into primary and specialty care teams and practices. Drawing on results from practice interviews and focus groups, the case study shares key insights and lessons learned from the perspective of providers, staff, and CHW/Ps. While the program is in the pilot phase at the time of publication, there is ongoing evaluation from the program's initiation, which has allowed for continuous learning. This case study focuses on a subset of the CHA programs at one medical group in California across five practices. The case study identifies facilitators and barriers of the program's implementation and early impact to practices and patients from the provider, practice staff, and CHW/P perspectives.

## Background

Blue Shield reviewed the evidence base for CHW/Ps and began its own program called the Community Health Advocate (CHA) program. Blue Shield adopted the “CHA” title for CHW/Ps to introduce roles within the enterprise and provider partners. Blue Shield first introduced CHAs to the Blue Shield Promise Health Plan (a Medicaid managed care plan) in 2018, with 11 CHAs who served over 4,200 Blue Shield Promise Medi-Cal members by supporting at-risk members with accessing care and addressing health and social disparities.

Blue Shield later launched the CHA pilot program across the state in partnership with multiple medical groups and community organizations, as part of the comprehensive Health Reimagined initiative aimed at transforming the healthcare system.^3^ As of January 2021, there are 17 CHAs in the Health Reimagined CHA pilot program. The CHAs are integrated into 10 primary and specialty care practices across four regions in California, including six practices from Hill Physician Medical Group^4^ in Sacramento County, three practices in Monterey County, one practice in Butte County, and one practice in Los Angeles County. Blue Shield CHAs can serve all members at a practice, regardless of member health plan insurance coverage or carrier.

A key aspect of the Health Reimagined pilot program embeds CHAs within primary and specialty care practices serving patients using a payor-agnostic approach, meaning the pilot reaches the entire practice's patient population and is not limited to Blue Shield members. This approach is intended to increase access to social services and community resources, improve health outcomes, reduce medical costs, and improve overall patient experience. The pilot program includes a high-touch, personalized provider-embedded and field based-approach to bridge to community resources and integrate social needs in the care plan. The CHAs are an expansion of the care team with workflows built into the day-to-day operations.

*Patient population reached by the CHA Program:* Since the CHA Program's initiation in October 2019, 17 CHAs have served a total of 1,906 patients and created 3,602 referrals, screening their populations for social needs over 2,838 times. A summary of high-level CHA program data by region can be seen in Table 1.

**Table 1:** High-level data from the CHA program through December 2020 is outlined below.

**Metric**	**Sacramento county**	**Monterey county**	**Butte county**	**Los Angeles county**	**All regions**
# Of social needs assessments	2,327	426	67	18	**2,838**
# Of patients	~1,000	843	42	21	**1,906**
# Of referrals	2,332	1,170	71	29	**3,602**
Top referral type	Mental and behavioral health	Physical health	Transportation	Food assistance	**Physical health**

*17 CHAs have served over 1,900 patients and created 3,600 referrals. The top referral types vary by region, as each region has a diverse population with specific social needs. The program start dates and number of practices/CHAs vary by region, impacting total volume in some areas*.

*A patient may have more than one referral. The CHA program in Sacramento was the first to launch and has more CHAs than the other regions, which contributes to its higher volume of patients and referrals. Bold values are for totals across all regions*.

The CHAs serve a diverse patient population. Sixty percent of the patients connected with a CHA were female, and one-third identified as Latino/Hispanic. At initial connection to a CHA, one in three patients who were referred to a CHA reported “fair or poor” health when screened for health-related quality of life measures. Patients reported an average of 19 unhealthy days of the past 30 days, including 7.7 physically unhealthy days and 11 mentally unhealthy days.

The top social needs identified from screening patients include housing, access to health care, and unemployment. CHAs made referrals to appropriate community resources to help patients address their unmet social needs. The top referral types were physical health and access to health care, individual and family support, food assistance, mental and behavioral health, and housing and shelter. The types of referrals to resources vary by gender and race/ethnicity. For example, for women, top referrals were for access to health care and mental/behavioral care. For Black/African American patients, the top referral type was for mental/behavioral health, and for Latino patients, the top referral type was for access to health care.

The top referral types also varied by practice type. Overall, Mental and Behavioral Health was the most prevalent among primary and specialty care practices. Primary care also had more physical health-related referrals, while specialty care practices had more referrals related to basic needs, such as housing and shelter, and individual and family support. The top referral types varied within each specialty care practice type, as shown in Table 2.

**Table 2:** The top referral types varied by practice type.

**Practice type**	**Top referral #1**	**Top referral #2**	**Top referral #3**
**Primary care**	Mental and behavioral health	Physical health	Benefits navigation
**Specialty care**	Mental and behavioral health	Housing and shelter	Individual and family support
Ob/gyn	Mental and behavioral health	Housing and shelter	Clothing and household goods
Pulmonary medicine	Mental and behavioral health	Benefits navigation	Utilities
Orthopedic	Transportation	Food assistance	Benefits navigation
Endocrinology	Physical health	Wellness	Transportation

*Mental and behavioral Health was most prevalent among both primary and specialty care practices*.

### Recruitment Process

Recruiting CHAs from the communities they serve brings significant benefits for the patient population because those individuals are more likely to have an intimate understanding of the socioeconomic factors faced by fellow residents. Evidence shows the importance of recruiting CHAs who can cross cultural and language barriers to bridge the gaps between providers and communities, and ultimately address key social determinants of health (13). CHAs must be able to connect to their neighbors and translate their interventions and messages in a way the community can understand. Qualifications of a successful CHA are focused on knowledge of the community, personality, and communication skills, rather than technical abilities (14). CHAs must guide their communities to realize their potential to create opportunities to achieve better health and well-being (15).

*Approach*: Blue Shield has a recommended recruitment process to ensure the most qualified individuals are selected for the CHA role (i.e., credibility in the community, language(s) spoken, rapport with organizations, ability to navigate community systems, etc.). Blue Shield's hiring team created the job descriptions medical groups use in their recruiting efforts. A sample job description can be found in Section 1 of the Supplementary Material. The recruitment process is led by the medical group's hiring team and management. Interviews are conducted by the medical group, who evaluate candidates based on their resumes and a Blue Shield evaluation form, make a final decision on who to extend offers, and finalize all pre-employment requirements.

### Training and Education

CHA training programs vary in content, focus, education, rigorousness, and time. One limitation to implementing a CHA program in California is the lack of state-level or industry standards for industry preparation (16). Most training programs are focused only on the development of skills that are needed in a very specific setting and cannot be widely scaled. Because of the lack of consistency and clarity in training, barriers exist to scaling CHAs within health care, public health, and social services settings on a state and national level (17). Several organizations, such as the California Future Health Workforce Commission, have developed plans of action to address the challenges related to supply, diversity, and geographic distribution of CHAs in primary care, prevention, and behavioral health settings. The Commission's recommendation is to scale the engagement of CHAs through certification, training, and reimbursement mechanisms (17).

*Approach*: To address training needs in our state-specific context, the Health Reimagined CHA Program partnered with experts in the field who are familiar with training processes for CHAs across the state. Blue Shield worked in collaboration with various subject matter experts and community-based organizations, such as Partners in Care Foundation^5^ and Rush University Medical Center – Center for Health and Social Care Integration^6^ to co-develop and deliver curriculum for the CHA program. CHAs from current cohorts or key partnerships serve as subject matter experts in their field and participate in the training process for future CHAs. Blue Shield also worked with HealthBegins^7^ to provide training to our provider practices on how to address social determinants of health in our member population. More insight into Blue Shield's external partners can be found in Section 2 of the Supplementary Material.

#### Blue Shield CHA Curriculum

Blue Shield engaged with Partners in Care Foundation as a training vendor with years of community-based experience to recruit the right individuals and share meaningful training aimed at refining CHA interactions with patients. The curriculum is designed to be a universal standard for all CHAs and includes various training modules that can be translated to any health setting. Racial justice and equity are themes interwoven throughout the curriculum and in ongoing trainings. CHAs complete training topics specifically focused on equity and inclusion in healthcare. The curriculum also incorporates an understanding of the racial/ethnic disparities in communities and how the CHA role aims to bridge the gap that exists between social and health disparities observed in the community with the care provided by the medical system. The curriculum is currently being used outside of Blue Shield and will be freely available for all Community Colleges in California. The curriculum includes CHA core competency training, behavioral and mental health, field training, and one-on-one mentoring. The mentoring aspect will resume post-COVID-19.

- The Blue Shield CHA training is 40 h of in-classroom instruction and 16 h of mentoring/shadowing in the community. The training curriculum addresses core roles/responsibilities and core competencies, with an emphasis on social justice, cultural humility, motivational interviewing, mental health, and health/chronic conditions, that are essential skills for CHAs to perform duties that include:
  - Assessment of social determinants of health needs for patients, documenting assessment results, educating patients, and communities, referring patients to community-based organizational resources to close unmet social needs and address health inequities
  - Active engagement, building rapport, establishing a trusting relationship, and continuous transparent communication with patients and their family support systems
  - Assisting patients with problem-solving barriers to health conditions by identifying, locating, connecting to, and navigating needed community and medical system services, including visiting patients at their homes, accompanying patients to medical and related appointments, and assisting patients with completing forms to access needed services.
  - Documenting activities and progress notes in appropriate systems and providing reports to management and providers.
  - Identifying gaps in community resources and medical systems and supporting the implementation of new solutions or services to close identified gaps through advocacy and education
- The Blue Shield CHA Behavioral Health training provides an additional 24 h of in-classroom instruction with a focus on behavioral health to support community members (mainly uninsured/underserved/migrants/homeless communities). The behavioral health training included background information on several mental health conditions, including depression, anxiety, PTSD, substance abuse or misuse, and cognitive function, among others. CHAs were also introduced to trauma-informed care and approaches to incorporate into their duties.

### Integration Into Teams, Provider Practices, and the Community

Evidence shows that CHAs have maximum impact when they are fully integrated into a care team by having defined roles and expectations, clear reporting and documentation structure, and mutual respect with supervisors and clinical staff (17, 18). These components ensuring optimal success for CHA integration into clinical teams are outlined in this section.

*Approach*: Prior to CHA implementation at the medical group's practices, Blue Shield carefully selected the practices in distinct regions across the state of California. Practices shared unique qualities that made them ideal to partner with for piloting solutions based on readiness and other factors (including rural/urban, specialty/primary care, large/small practice, etc.). This process involves evaluating the potential sites against baseline criteria and researching the current standing with the partner. Blue Shield presented the program and confirmed the interest of each provider practice, then defined shared goals and objectives of the practices to ensure the success of the CHA program. Following this initial engagement, a readiness assessment was conducted to determine viability for the CHA program. All sites in this study indicated a need for CHAs at their practices.

#### Tools and Infrastructure to Support CHA Integration

Documenting data on the CHAs daily activities is a key aspect of successful integration into clinical settings. The workflows of a CHA program are strongly influenced by integrating patient medical records with documented interactions between CHAs and their patients. This can be done by implementing a user-friendly, community-based platform that centralizes these functions (19). Documentation has proven instrumental to monitor interventions, provide feedback to CHAs and their supervisors, and identify training needs for the future (20).

Blue Shield leveraged tools and infrastructure to help facilitate the training and implementation of CHAs as described above. Meaningful data from the tools used to support CHA infrastructure was utilized in the evaluation to understand the impact of the CHAs in the program. The CHAs begin by engaging with the patient, then, screening for social needs using a social determinants of health screening tool called the Protocol for Responding to and Assessing Patients' Assets, Risks, and Experiences (PRAPARE)^8^. From the initiation of engagement, they begin building rapport with the patient as they co-develop a care plan during home visits, or telephonically, to identify the patients' goals and interventions required to achieve these goals. Upon identifying needs or gaps in a patient's care, the CHAs make referrals to community resources to address health and social needs. Additionally, they continue to work with the patient and community to support the implementation of new solutions that ultimately improve community health.

Blue Shield leverages a tool called mySidewalk,^9^ which allows CHAs to identify social risk factors of communities and conduct rapid community health needs assessments by creating customized reports and dashboards. Blue Shield collaborated with mySidewalk to develop The Neighborhood Health Dashboard,^10^ which allows CHAs, providers, and all other Californians to create customized health reports on community strengths and needs. The Department of Health Care Services (DHCS) recently awarded Blue Shield the first place Innovation Award for the Neighborhood Health Dashboard.

The second tool, Unite Us,^11^ is used to facilitate bidirectional referrals across an array of service types, and to support prevention, early identification and treatment of individuals' top priority health concerns and social needs. The platform tracks the number of patients a CHA connects with, the social needs identified through the PRAPARE screener, responses to the CDC Healthy Days questionnaire, and referrals created to address the patient's social needs. Research has shown the effectiveness of referral systems that provide an important link from the community to the broader health system (21).

#### Provider and Clinical Staff Training

In addition to training the CHAs, it is also important to inform the provider and clinical staff on how embedding a CHA in the medical group practice will impact their work. Sharing information on the CHA role, the types of social needs observed in the community, and the capabilities of the CHA with the provider and clinical staff is critical for the success of the program. The CHAs give voice to the patients' social needs and provide a new lens on the lives of their patients for the providers and clinical staff to see. Blue Shield encourages the practices to discuss the CHA program's aims and capabilities with all staff at the practice, and include the CHAs in these meetings where the CHA can share key aspects of their work. CHAs translate data to action by obtaining insight into local community factors with the use of mySidewalk and Unite Us. The CHA can share data and key insights with the providers and staff on social needs that are unmet and discuss how this can impact patient health.

#### Team Huddles and Enhancement

Blue Shield recommends the providers and clinical staff engage in regular team meetings to ensure the CHAs have the information they need to succeed, and their patient population receives appropriate services to address their needs. Studies have demonstrated the value of frequent team meetings to discuss progress and outcomes of patient interventions, or to review CHA roles and training (22). The approach for the Blue Shield CHA program is to include the CHA in team huddles with the practice to discuss care planning, 360-degree information sharing, and identification of resources and barriers for the patient. The CHAs share their external subject matter expertise of community resources and extend the information to the practice.

## Methods

**Figure 1:**
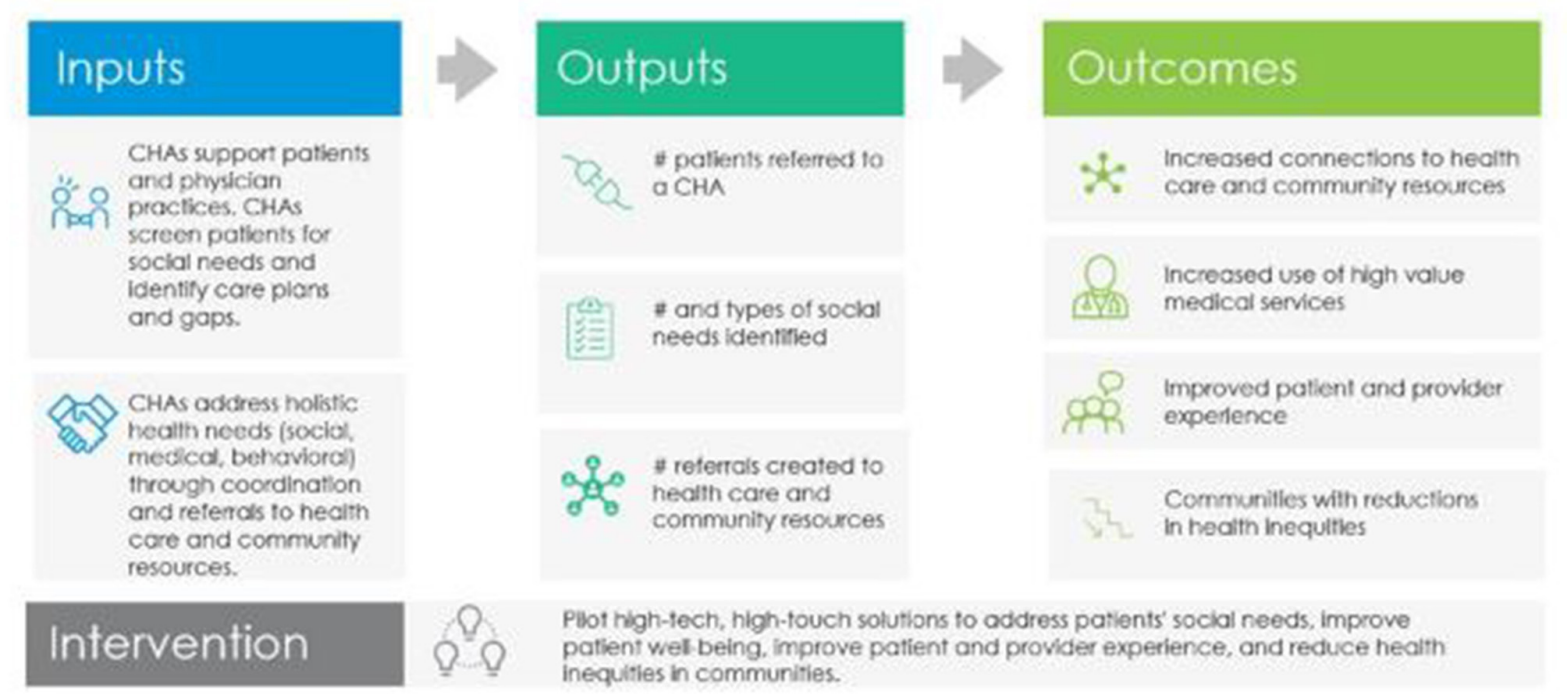
Logic model for blue shield's CHA program.

Evaluation and quality improvement are integrated into the CHA program pilots that run from 2019–2021. The evaluation approach uses the Center for Disease Control's Program Evaluation Framework to incorporate standards and phases that are “useful, feasible, ethical, and accurate” (23). The goal is to have a process of continuous and systematic learning with the pilots. Stakeholders are engaged and part of program improvement from the beginning of implementation. Blue Shield's CHA program logic model (Figure 1) illustrates how the interventions are designed to yield desired outcomes.

Blue Shield uses a mixed-method approach and incorporates qualitative and quantitative data collection throughout the CHA program's two-year pilot period. Quantitative data collection is ongoing, which includes the number of patient cases opened and closed, social needs identified, and types of referrals to resources. Additional quantitative data is being collected to capture the provider and practice perspectives on the CHA program *via* a practice survey.^12^ This survey is currently being administered and some practice responses are pending. Thus, this case study focuses primarily on qualitative results from interviews with providers, practice staff, and CHAs. Qualitative data methodologies are described in additional detail below. This project was conducted for the purpose of Blue Shield's evaluation and quality improvement operations and does not meet HHS's definition of research.

### Qualitative Data

Research shows that engaging CHAs in the evaluation process is essential, their unique perspectives and roles strengthen the evaluation efforts and help identify unmet community needs (24). To date, Blue Shield has conducted two focus groups with five of the CHAs included in this case study. The first focus group took place approximately 3 months after their start date in January 2020 and included four CHAs embedded at four different practices. The second focus group consisted of five CHAs and took place in September 2020, about 11 months after their start, which included the original four CHAs. Each focus group lasted approximately 90 min. Interview domains included training and onboarding, integration into the practice and care team, their experience screening and referring patients for social needs, and their impact on the practice and patients. The CHA interview guide can be found in Section 3 of the Supplementary Material.

Approximately 9 months after the CHA program was implemented, Blue Shield staff conducted 10 semi-structured interviews with a total of 18 providers and office staff at five primary care and specialty practices where the CHAs are embedded. Interviews were conducted telephonically over a two-week period in June 2020, and each lasted ~ 60 min. Interview domains included provider and staff experience with the CHA program, preparation for integrating the CHA into the practice, impact on the practice and patients, and understanding of the CHA role and social needs of the practice's patient population. The provider/staff interview guide can be found in Section 4 of the Supplementary Material.

Participation in the focus groups and semi-structured interviews was voluntary. The evaluation is conducted for the purpose of Blue Shield's usual qualitative improvement operations. All participants are informed of the goals of the evaluation, what their participation will involve, and how their information with the CHA program will be used for quality improvement.

#### Data Analysis

In each focus group and interview, two Blue Shield team members were present, one led the conversation and the second took detailed notes. Recording was not available and thus the detailed and summary notes from the Blue Shield staff were used for analysis. All qualitative data was de-identified and aggregated for analysis. Using qualitative thematic analysis all data was coded using QSR NVivo software to identify themes that highlight provider, staff, and CHA perspectives on the impact of the CHA joining the practice.

Five members from the Blue Shield team collected, coded, and analyzed the data from the provider/staff interviews and CHA focus groups. Two team members created initial code books based on objectives of the pilot and core themes of interest, one for the CHA focus groups and one for the provider/staff interviews. To align on definitions and use of the codes, all five team members coded one provider/staff interview. The team discussed differences and aligned on definitions. The remaining interviews were each coded by two team members. The same process was followed for the CHA focus groups. If a new theme was identified through the coding process, the team discussed the potential new code and added if consensus was reached. Prior interviews were re-coded to incorporate the new codes. Codes were then organized into higher level concepts. The team reviewed the emerging concepts and identified themes. Findings were summarized and learnings were shared with stakeholders, including CHAs, providers, and practices. Stakeholder input facilitated the ability to confirm findings and develop actionable learnings for program improvement.

## Findings

### Recruitment Process

Blue Shield found that having a direct collaboration with the medical groups in the recruitment efforts resulted in streamlined communication and yielded successful recruitment results. The candidate pool was diversified, and all candidates considered were well-qualified. The effectiveness supported the overall hiring goal because it was efficient enough to deliver high-quality, more engaged hires that led to a competitive advantage that directly impacts the program. Additionally, the resulting CHA workforce is diverse and shares life experiences with the patients in the communities they are assigned to serve.

### Training and Education

The Blue Shield CHA curriculum is a compendium of materials from partners, subject matter experts, and several publicly available sources. The CHA curriculum is critical to ensure CHAs are adequately trained and supported so they can be effective in their roles of supporting patients around social needs. The curriculum and training approach is meant to be comprehensive and includes core competencies around social needs and social determinants of health, an introduction to behavioral and mental health tools, mentorship, and one-on-one training. With holistic training, the CHA is able to improve access to health and community services for patients, contribute to community development, and ultimately impact social determinants of health in the local community. CHAs have reported high satisfaction with the content and depth of the training. The CHAs receive continuous training every quarter further supporting the educational material introduced in the curriculum as well as creating opportunities for CHAs to share best practices with other CHAs.

### Integration Into Teams, Provider Practices, and the Community

#### Interview Findings

In the 10 interviews with providers and staff and the two focus groups with CHAs, interviewees described their perspectives on the role of a CHA, and what factors helped enable his or her success. Key themes are described in this section and are outlined in Table 3.

**Table 3:** There were six key themes from provider, staff, and CHA interviews and focus groups on the perspectives of the CHA role, and what factors enabled his or her success.

**Themes**	**Quotes from practice staff**
**CHAs with shared life experiences as patients**	“*I have experienced working with the patient and the patient is hesitant to tell me their social needs (female CHA talking about male population). Machismo is very established and I'm a 5'1 female, every time I have a new male patient referred by a male friend I'm surprised.” – CHA “When you are looking for a health advocate, I think diversity is really important. And that really expands the outreach to different patients, depends on their ethnicity, their language. The CHA helped me a lot to reach out to patients where language was the biggest issue.” – Provider “Our CHA has a social services background, and a military background. He has had much success connecting patients to Veteran services. He has experience with homeless populations, and he has had much success with housing, and food services.” – Practice staff*
**Establishing role clarity at the practice**	“*There was not a lot of information for all staff at the practices on the role of the CHA prior to implementation*” and “*the first day I showed up and the clinic staff didn't know what the position was.”– CHA “For me, it felt nebulous because we weren't totally sure how to use the CHA. What the actual role would be.” – Provider*
**Increased understanding of patient populations' social needs**	“*This (CHA) is one of those things I didn't really believe in. I was not an enthusiastic adopter. I was foolish. I thought people were doing better than they are, and I was wrong.” – Provider*
**Primary Care vs. Specialty Care Practices**	*N/A*
**Improved patient engagement with their health and care**	*CHAs “give the patients a lifeline and a personal number they can call.” – CHA “What [CHA] has done is go into the community and find the resources that are already there, but they are different bodies not really connected to each other. She went to a place where resources were available, she brought those resources into my clinic…the doctor is telling them to eat healthy, now this CHA is telling them where they can get these foods.” – Provider*
**Impact of COVID on social needs and CHA response**	“*Whatever routine the patient had before has mostly been put on hold. Causing tremendous stress.” – CHA ”We want to create a trusting relationship and because of the relationship (with the CHA) we've mitigated the need for some patients to be hospitalized." – Practice staff*

#### Shared Life Experiences

The CHA Program intentionally hired a diverse CHA workforce that has shared life experiences with the communities they serve (e.g., CHAs with language competencies or who are members of the community). Providers and the CHAs have noted the importance of having CHAs who understand and can relate to their patients and cite this as a key component of the program's success.

Shared life experiences or characteristics can help CHAs establish trusting relationship with their patients that has often allowed them to overcome social or cultural barriers with patients. As one CHA recalled, “*I have experienced working with the patient and the patient is hesitant to tell me their social needs (female CHA talking about male population). Machismo is very established and I'm a 5'1 female, every time I have a new male patient referred by a male friend I'm surprised.”* This CHA understood the nuances of gender expectations that existed with her Latino patient, yet the trust and rapport that she patiently established with the patient provided an opportunity to support a patient that may not otherwise have engaged with the practice. Another CHA found that sharing her own story around a local event that affected the community helped patients see her as someone with a deep understanding of the community. The ability to build trust is important with patients, as providers noted that patients may not always ask for help.

The providers and staff highlighted several instances where the CHA's shared background or life experiences (e.g., military service, language) enabled the CHA to reach a broader patient population. The shared background, coupled with the additional time the CHA can spend with a patient, allows them to create a connection with the patient beyond what the provider could achieve.

The findings are similar to what other studies have shown (3), that understanding the culture and practices of the communities where CHAs reside is essential to implementing solutions to improve the health of community members. Health care providers serving diverse communities often lack an understanding of their community's language, culture, and history, creating gaps in effectively treating marginalized communities (24). Being embedded in the community also allows CHAs to work alongside their patients more conveniently, and increases their ability to assist those who have difficulty accessing care (13).

#### Establishing Role Clarity

CHAs report that it takes time to develop good working relationships with providers and practice staff. Some CHAs noted that when they began in their position, other practice staff seemed unaware of the purpose of their role, or how to interact with them. As one CHA said, “*there was not a lot of information for all staff at the practices on the role of the CHA prior to implementation*” and “t*he first day I showed up and the clinic staff didn't know what the position was.”* In some cases, only practice leadership were familiar with the CHA's role, and other practice staff were not, resulting in uneven utilization of the CHA's services. Most CHAs said that after several months, relationships improved, as other staff members became more familiar with the role.

Providers and practice staff agreed in interviews that there was an opportunity to further communicate the capabilities of the CHA with the full practice. The CHA role was new at all practices and for some providers having a CHA “*felt nebulous because we weren't totally sure how to use the CHA. What the actual role would be.” One* provider who was actively involved in the onboarding of a CHA said he did not promote the CHA enough to clinic staff at the start of the program and as a result, the practice was not using the CHA to their full capacity. The provider states, “*I assumed that when I told people about and introduced her and gave her a lot of verbal support “hey you guys should try this” I thought if I did that once or twice it would be enough.”* Clinic staff had similar feelings to the providers and felt that “*people gravitate towards those in charge and don't focus on other members of the team, who may not be involved in practice leadership.”* Improving adoption of the CHA was dependent on clearly establishing activities of the CHA role, ensuring that all providers and staff at the practice were aware of the CHA, and continuous communication throughout the practice.

Practices that established clear onboarding processes –including introducing the CHAs to the entire staff— reported that integrating the CHA into the practice went smoothly. One practice also emailed all patients to introduce the CHA and highlight how they could support patients. This multi-channel communication strategy helped connect CHAs, practice staff, and patients from the beginning.

Practices reported that CHAs were increasingly effective over time, as they continued to carve out their niche in the practice infrastructure. During the COVID pandemic, CHAs have been integral to supporting patients, as social needs have increased, and available resources have changed.

#### Understanding Social Needs

Providers often underestimate the level of social needs of their patient population. After having a CHA in their practice, providers consistently note the success of the program and the increased ability of the practice to care for their patients because the CHAs help the entire care team to better understand and address patients' social needs. In the current COVID environment providers recognize the need for CHA services. As one provider mentioned, “*Many people live alone, and don't have family nearby and have lost their social support.”* Some providers believe that the CHAs are time savers and improve quality of care provided; “*the CHA is greatly improving the care we can give to our patients.”*

Several providers initially reported they did not believe their practice needed CHA services. However, after the CHA was integrated into their practice, the providers reported appreciating learning more about their patients' social needs. For instance, one provider had been seeing a patient for 10 years and did not realize the financial burden the patient faced as a result of weekly dialysis until the CHA brought it to the provider's attention. Another provider said “*this (CHA) is one of those things I didn't really believe in. I was not an enthusiastic adopter. I was foolish. I thought people were doing better than they are, and I was wrong.”* CHAs contribute to the provider and practice a new or different perspective of their patients, sharing a window into the patient's life that the provider might not otherwise see, but that can impact the patient's health or care.

#### Primary Care vs. Specialty Care Practices

CHAs were successfully integrated into Primary Care and Specialty Care practices in the CHA Program. Across the five practices, there was no clear pattern in facilitators or barriers to embedding CHAs by practice type. Some practices had more bandwidth related to time and staff that facilitated implementing the CHA program and were able to embed the CHA more quickly than other practices. Additionally, while all practices had a provider champion, there were varying levels of readiness and receptiveness by the practice to addressing social needs for patients via the CHA program. Some provider champions were more proactive in communicating the importance of social needs and the CHA to the rest of the practice, which enabled all providers and staff to incorporate the CHA role into the practice workflow, care team, and the broader culture. For the provider champions that were less engaged in communicating the importance of the CHA role, integration into the practice culture and care team took longer.

For both primary and specialty care practices, behavioral and mental health was among the most pressing social needs, which coincides with the COVID-19 pandemic. Among the specialty practices, the CHAs were able to tailor their support around specific populations or needs within a smaller scope. For example, a CHA at a specialty care practice coordinated classes with a community-based organization focused on diet and food for diabetes care management. The specialty care practice had an engaged pool of patients with diabetes that benefited from the support of the CHA. In specialty practices, the CHA was often able to focus their support and scope based on the specific needs of the patient population. At primary care practices, the scope of social needs was broader, and the types of support needed by patients varied. In primary care practices, the top referrals to resources were for mental and behavioral health, such as individual counseling, benefit navigation, housing, and transportation. For specialty care practices, the top referrals to resources varied by specialty type. For example, the CHA at an obstetrics and gynecology practice had the highest referrals for mental health, support groups, and housing, while a CHA at an orthopedics practice had the highest referrals for ride coordination, emergency food, and job search. In each of these examples, the CHAs in specialty practices supported patients around social needs associated with the care and management most needed by the specific patient population, whereas the CHAs in primary care practices had more diversity in the types of social needs and the resources they engaged with to best support their patient population.

#### Patient Engagement With Their Health and Care

Patients feel empowered by the CHA program and are starting to treat their healthcare differently. CHAs report that some patients have learned to advocate for themselves and are able to ask providers and community organizations for resources at the recommendation of the CHA. With help from the CHA, patients have become more comfortable using the mediums of communication from their providers, such as the provider's online platforms, and are now more engaged with accessing services and communicating with providers.

CHAs build trust with their patients by sharing their own stories and finding commonality in their experiences with patients. In this way, CHAs can often learn about a patient's needs that a patient would not have otherwise shared with a provider. As one CHA mentioned, CHAs “*give the patients a lifeline and a personal number they can call*.” The trust between a CHA and a patient can translate to improved relationships between patients and providers. As one CHA recalled the story of a homeless patient whom he had supported with accessing housing, the patient now saw the practice as his family and came in several times a month to socialize.

For providers, the CHA can support the care and recommendations a provider gives a patient. At one provider's practice, the CHA partnered with a community-based organization to bring nutrition classes for patients into the provider practice. The CHA can help patients put into action the provider's care plan. As the provider noted “*the doctor is telling them to eat healthy, now this CHA is telling them where they can get these foods.”* As the CHA supports the find resources to address the patient's needs, the CHAs also help the patient translate the provider's recommendations, allowing the patient to more fully engage with their own care.

#### COVID Impact

During the COVID pandemic, CHAs have been critically important in supporting patients, as social needs have increased, and available resources have shifted. Some providers report their patients are experiencing increased angst, drug/alcohol abuse, domestic violence, decreased physical activity, vaccine confusion and hesitancy, along with stresses from lack of school consistency, loss of jobs and inability to pay for rent, food, and transportation. The additional limitations in support systems, in combination with the above, have also led to role shifts — with some CHAs providing more of a counselor/support role, on top of their roles screening and provider referrals for needs. One CHA described the fear and stress their patient was encountering because of the pandemic and the changes in accessing health care: “Whatever routine the patient had before has mostly been put on hold. Causing tremendous stress.” When supporting patients in quarantine because of COVID, staff also expressed how the CHAs deescalated the fear and stress of being in isolation and reduced unnecessary hospitalizations: “we want to create a trusting relationship and because of the relationship (with the CHA) we've mitigated the need for some patients to be hospitalized.” As patients shifted to virtual care or rescheduling procedures and appointments, CHAs helped to coordinate care for patients and provider overall support during the process.

CHAs also described the changing availability of resources for their patients as COVID impacted the availability and funding of all sectors, including the local organizations that patients could be referred to. CHAs had to do additional outreach to resources and think creatively to address patient's social needs.

## Discussion

Similar to previous research (18, 25, 26), Blue Shield found that the Community Health Advocate program improved care coordination, increased referrals to resources, and improved support of social needs of patients. Results also found that CHAs can impact the provider and practice experience and assist providers in deepening their understanding of the social needs of their patients. Having this additional knowledge allows providers and staff to tailor their care to the patient's needs. Understanding the types of social needs that patients may have and are able to share in a clinical setting with the CHA can provide valuable information to providers and health plans, to better target the needs of patients and the community.

Blue Shield has successfully incorporated CHAs into primary care and specialty practices and to those serving mainly commercial patients. The CHA program effectively balances its standardized high-quality didactic and experiential training, while allowing flexibility to adapt training to various practice types and workflows. The CHA program contributes valuable insights into creating and implementing a payer-agnostic, provider-embedded model.

While all provider practices found the CHA beneficial to their patients and practice, some had challenges ensuring the full practice understood the role and capabilities of the CHA. There was no observable difference by type of practice, i.e., primary vs. specialty care. Slower adoption of the program and integration of the CHA into the care team differed by practice bandwidth (time and staffing), level of engagement by the provider champion, and overall culture of the practice around the importance of social determinants of health. Discussions are underway with subject matter experts, Blue Shield, and providers and clinical staff to provide detailed education and communication of the CHA program at future implementations and to share best practices from the initial providers. This includes clear communication of the CHA role and abilities, change management among the practice to institute effective workflows and increase communication between all providers, staff, and the CHA, and increase awareness of social determinants of health and the link to health care. To address some challenges with data sharing and communication between CHAs and practices, Blue Shield is maximizing the technology and resources CHAs use in their day-to-day work and partnering with providers and medical groups to align on data sharing and access.

### COVID-19 Response

The pandemic has exposed and amplified long-standing systemic health and social inequities along with the associated increased risk of infection, hospitalization, and death in marginalized racial and ethnic groups^13^. The impact of COVID-19 on both the CHAs and patients had to be addressed in the CHA program efforts. Several standard protocols in the CHA workflow were adjusted to fit the needs of members and CHAs to accommodate COVID-19 pandemic precautions. CHAs moved predominantly to telehealth solutions as alternatives to home visits, except under situations where telehealth would not adequately address member needs. CHAs also administered a unique COVID-19 screening assessment tool developed by Unite Us in place of the PRAPARE screener to evaluate patient social needs at risk or impacted by COVID-19. Additionally, CHA role flexibility allowed some practices to leverage CHAs using telehealth to help bridge the impacts to access to care when the pandemic first began. CHAs were able to provide support to patients who were experiencing stress and fear during the pandemic, by assisting with telehealth use, coordinating care, finding resources for new social needs, and being a source of emotional support for patients. In response to the availability of changing resources, Blue Shield made emergency funds available to the CHAs for immediate need or if no resources were available.

#### Healing Circles

In response to the added stresses and uncertainties of the COVID-19 pandemic, Blue Shield has implemented Healing Circles, offering them to all CHAs. Healing Circles help people step out of their day-to-day and into a safe and accepting environment where they explore ways of healing. They provide a safe place to discuss the impact of the recent events on the CHAs themselves, their patients, and families, and provide an environment for CHAs to decompress emotionally and mentally. They also provide ways for CHAs to learn to support each other, and practice self- and stress-management tools. Healing Circles have been shown to enhance camaraderie and support for practitioners who work with patients with significant needs (27). They have also resulted in statistically significant improvements in quality of life and emotional relief for participants (28).

### Future State of CHA Program

Blue Shield values the work of CHAs and recognizes the unique role they play in health care. Blue Shield intends to remain a leader in promoting this work for members, the communities where they reside, and scale similar programs on a broader level so other communities can benefit from this work.

#### CHAs and Statewide Implementation

A statewide Community Health Advocate Program has the potential to maximize positive impact in addressing Blue Shield members' unique social needs, while improving social determinants of health, and ultimately producing a sustainable, affordable, and equitable reduction in high-cost unnecessary utilization costs. Blue Shield is launching a pilot for Blue Shield members in specific regions to commence the statewide approach. CHAs will serve and conduct telephonic outreach to members at risk of having a need, as well as high-risk members with care gaps, complete a psychosocial assessment, and telehealth solutions as alternatives to home visits. This will allow Blue Shield to proactively reach patients to be connected to CHAs without having to be referred by a provider.

#### Targeting Populations

Building on lessons learned from the initial experience with the CHA program to date and considering ways to address racial inequities, Blue Shield is developing pilots for targeted populations that could benefit from a CHA program. One example is racial inequities in maternal and infant health. According to the US Department of Health & Human Services Office of Minority Health, Black women are three–four times as likely to die from pregnancy-related causes than non-Hispanic White women, and Black infants have over two times the infant mortality rate as non-Hispanic Whites^14^^,^,^15^. Blue Shield acknowledges the perpetual racial health inequities that directly impact Black mothers and infant maternal health, and is piloting initiatives to remove social barriers and disparities seen in Black maternal and child health outcomes. One of these initiatives is the addition of doulas to the Community Health Advocate program to improve pre- and post-partum support systems, improve member access to critical maternal care services, improve maternal quality of care, and address maternal behavioral and mental health.

#### Funding and Reimbursement Mechanisms

Part of the evaluation of the CHA program includes the consideration of the financial and social returns for health plans, providers, patients, and community partners. Funding CHAs is a challenge that has been widely identified, as providers or practices alone may not be able to carry the financial burden of funding a CHA role (29, 30). Blue Shield is exploring various CHA program models to ensure the continuity of the CHA role and expansion of the workforce across California and nationwide. Blue Shield is also looking at reimbursement mechanisms similar to nationwide Medicaid programs.

### Limitations

This case study of the CHA program presents initial data from the provider and CHA perspective and is limited to early learnings of the program. Future work will include additional data sources to complement the current findings, such as completed practice surveys, aggregated patient health outcomes, and Blue Shield member utilization and cost of health care. Additionally, this case study focuses on a subset of the CHA programs at one medical group, thus generalizability to a broader population is limited.

The payor-agnostic model of the CHA program presented challenges to implementation, specifically around fidelity of the model as Blue Shield did not have full control of implementation at the practice level. Additionally, the internal evaluation is limited due to the ability to access only Blue Shield members' health care utilization information. Blue Shield does not have access to patient specific outcome data for non-members, thus limiting evaluation of the full patient population at the practices, and as a result, the full impact of the CHA program.

The COVID-19 pandemic has impacted the CHA program in multiple ways, such as exposure to the virus among patients and staff, protocol changes to the CHAs workflows and changes to their scope of work, and drastic limitations to in-person care. Each practice site put in different protocols to address the pandemic, resulting in greater variations to the CHA program than anticipated.

The challenge of sustainability beyond a health plan's pilot program is similar to that of other organizations that struggle with funding CHA programs. While CHA programs have been shown to benefit providers and patients, ensuring proper funding for CHAs remains an ongoing challenge that requires significant statewide and anchor organization (including CHW organizations) partnerships to resolve.

## Conclusion

The Blue Shield CHA program continues to address the gaps in recruitment efforts, training and education, and successful integration of CHAs into teams, provider practices, and the community. The program saw positive outcomes from recruiting a diversified pool of applicants, as it supported the delivery of high-quality candidates that directly impacted the program's success. Blue Shield's thorough CHA training curriculum, co-developed with subject matter experts in the field, proved critical to improving access to health and community services and ultimately impacting social determinants of health in the local community. Interviews with all stakeholders around the integration of CHAs into provider practices and the community brought out several themes on what factors enabled CHA success. Providers found value in having access to a CHA workforce with shared life experiences of the communities they served. CHAs embedded in the care team also increased the provider and staff's understanding of the social determinants of health that impact a patient's health. Embedding CHAs in the care team also impacted the patients' engagement with their health and care and allowed them to be more comfortable communicating with providers.

While all provider practices reported successful outcomes of embedding CHAs in the practice, the CHA program continues to adapt to address challenges faced by all stakeholders. The COVID-19 pandemic has especially amplified the need to support patients, with increased social needs prevalence in the community. Blue Shield plans to remain a leader in promoting CHA work for all patients and the communities they reside.

## Data Availability Statement

The data supporting the conclusions of this article will be made available by the authors upon request and with approval from Blue Shield of California's outbound data process.

## Ethics Statement

Ethical review and approval was not required for the study on human participants in accordance with the local legislation and institutional requirements. Written informed consent for participation was not required for this study in accordance with the national legislation and the institutional requirements.

## Author Contributions

SC, LP, and JE contributed to conception and design of the program. ST and MA oversaw the data collection and analysis plan. ED, ST, BBe, RP, AL, and LM performed the qualitative analysis. CP and ED wrote the first draft of the manuscript. BBe, LM, ST, and RP wrote sections of the manuscript. BBo, JS, and AC served as subject matter experts and contributed to interpretation and analysis of the findings. All authors contributed to manuscript revision, read, and approved the submitted version.

## Conflict of Interest

The following authors are employee of Blue Shield of California: CP, ED, LP, RP, ST, BBe, AL, JS, MA, JE, and SC. AC is an employee and BB a provider at Hill Physicians Medical Group. The remaining author declares that the research was conducted in the absence of any commercial or financial relationships that could be construed as a potential conflict of interest.

## Publisher's Note

All claims expressed in this article are solely those of the authors and do not necessarily represent those of their affiliated organizations, or those of the publisher, the editors and the reviewers. Any product that may be evaluated in this article, or claim that may be made by its manufacturer, is not guaranteed or endorsed by the publisher.

## Footnotes

^1^Centers for Disease Control and Prevention. About Social Determinants of Health. (2021). https://www.cdc.gov/socialdeterminants/about.html (accessed March 15, 2020).

^2^Centers for Disease Control and Prevention. Integrating Community Health Workers on Clinical Care Teams and in the Community. (2020). https://www.cdc.gov/dhdsp/pubs/guides/best-practices/chw.htm (accessed March 15, 2020).

^3^Blue Shield of California. Health Reimagined. (2021). https://healthreimagined.blueshieldca.com/ (accessed March 15, 2020).

^4^Hill Physicians. (2021). https://www.hillphysicians.com/ (accessed March 15, 2020).

^5^Partners in Care Foundation. (2021). https://www.picf.org/ (accessed on March 15, 2021).

^6^Rush University Medical Center. Social Work and Community Health Services. (2021). https://www.rush.edu/services/social-work-and-community-health-services (accessed on March 15, 2021).

^7^HealthBegins. (2021). https://healthbegins.org/ (accessed on March 15, 2021).

^8^National Association of Community Health Centers. Protocol for Responding to and Assessing Patients' Assets, Risks, and Experiences. (2019). https://www.nachc.org/research-and-data/prapare/ (accessed on March 15, 2021).

^9^mySidewalk. (2021). https://mysidewalk.com/ (accessed on March 15, 2021).

^10^Blue Shield of California. Neighborhood Health Dashboard. (2020). https://healthreimagined.blueshieldca.com/neighborhood-health-dashboard (accessed on March 15, 2021).

^11^Unite Us. (2021). https://uniteus.com/ (accessed on March 15, 2021).

^12^Practice survey was distributed to Clinical Providers (MD, DO, NP, PA, RN) and Staff (office managers, administrative staff). Community Health Advocates did not receive the practice survey.

^13^Centers for Disease Control and Prevention. Health Equity Considerations and Racial and Ethnic Minority Groups. (2020). https://www.cdc.gov/coronavirus/2019-ncov/community/health-equity/race-ethnicity.html (accessed March 15, 2020).

^14^Taylor J, Novoa C, Hamm K, Phadke S. Eliminating Racial Disparities in Maternal and Infant Mortality. (2019). https://www.americanprogress.org/issues/women/reports/2019/05/02/469186/eliminating-racial-disparities-maternal-infant-mortality/ (accessed March 15, 2020).

^15^U.S. Department of Health and Human Services Office of Minority Health. Infant Mortality and African Americans. (2019). https://minorityhealth.hhs.gov/omh/browse.aspx?lvl=4&lvlid=23 (accessed March 15, 2020).
