# Supplementary material for: Adding a Seat at the Table: A Case Study of the Provider's Perspective on Integrating Community Health Workers at Provider Practices in California
Source: Front Public Health. 2021 Oct 28;9:690067. doi: 10.3389/fpubh.2021.690067 (PMC8581190; doi:10.3389/fpubh.2021.690067)
Supplement: Supplementary file 1 [file Data_Sheet_1.docx]

Supplementary Material

# Community Health Advocate Sample Job Description

#
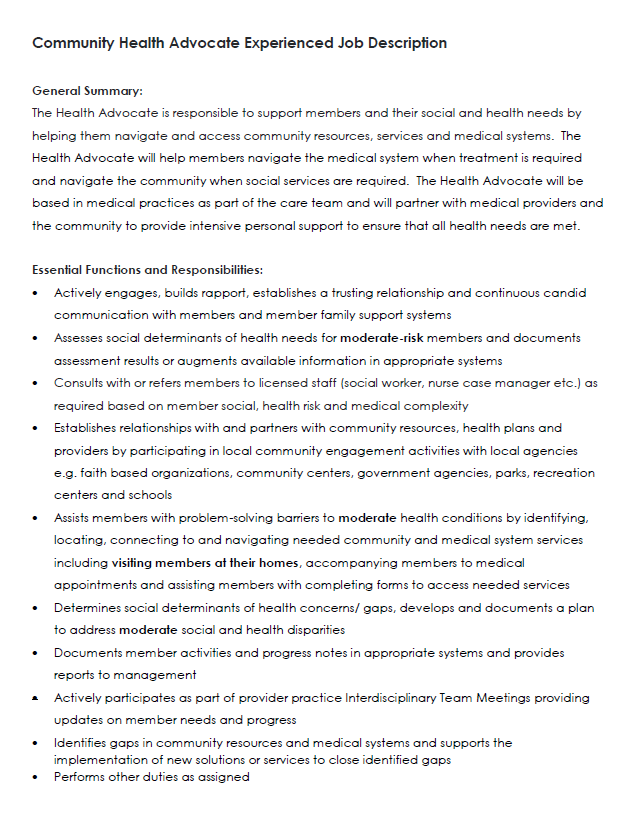


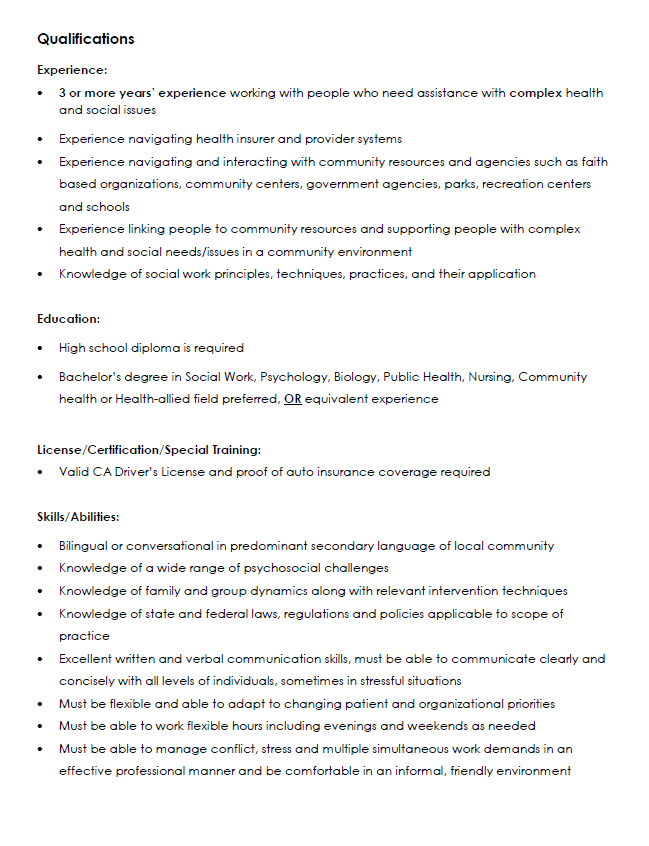


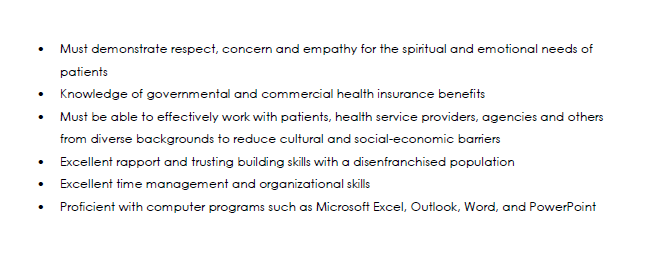


# Blue Shield of California External Partners

| **External Partners** | **Role in Blue Shield CHA Program** |
| --- | --- |
| Partners in Care Foundation | Partners in Care Foundation (PICF) shapes the evolving health system by developing and spreading high-value models of community-based care and self-management for diverse populations (PICF, 2020). Blue Shield partnered with PICF to develop the CHA curriculum. |
| Rush University Medical Center – Center for Health and Social Care Integration (CHaSCI) | CHaSCI at Rush University Medical Center (Chicago, IL) is an applied think-tank working to advance practices and policies that integrate community health workers into health care to break down barriers to health and enable equitable outcomes. Blue Shield partnered with Rush to co-develop and deliver the CHA curriculum. |
| HealthBegins | Blue Shield partnered with HealthBegins, a leading provider of training and education solutions for health care professionals, to provide meaningful training to our provider practices on social determinants of health. |
| Hill Physician Medical Group (HPMG) | Hire, supervise, and deploy CHAs in Sacramento County within the HPMG practice sites. |

# Community Health Advocate Interview Guide

**Community Health Advocate (CHA) Interview Guide**

**Introduction**

1. What does an average day (or week) look like for you?
   1. What types of activities make up your day?
   2. What activities involve direct contact with patients?
   3. How frequently do you interact with fellow health advocates?

**Integration into the practice**

1. How have your first couple months been at the practice?
   1. Is there anything that could have been done better to set you up in your position?

- *Probe: Training? Tools and other resources? Support?*

1. Did you have the resources and tools to do your job?

- *Probe: At the start of the position? Currently?*
  1. Anything that you still need or that would help you?

**Intervention Characteristics**

We’d like to understand more about your interactions with patients.

1. Can you tell me about how you are referred to patients?
   1. What works well?
   2. What can be improved?
2. Can you describe the workflow in assisting patients?

*Probe: What works well for you when working with patients? What is challenging when working with patients (case mgmt notes, screening, referring patients to resources?)*

1. What has helped you be effective in your role as a Health Advocate?
2. What has been challenging?
3. In your opinion, what is the biggest barrier to linking the patients with their needed resources?
   1. What would be most helpful to you to help them address these barriers?
4. What do you enjoy most about your work?

**Training**

1. Do you feel you were prepared to begin your work as a health advocate?
2. Have the skills you learned during training been valuable to you now that you are on the job?
   1. Are there additional trainings that you would like to have access to?

**Patient impact**

1. In your opinion, do patients have increased knowledge of their conditions and available resources?
2. What do you think patients find most helpful from your support?

**Conclusion**

1. Do you have any success stories that you’d like to share, especially around patient care or building relationships with community organizations?
2. Is there anything we have not asked you about today that you would like to share?

# Provider and Staff Interview Guide

**Provider and Practice Staff - Interview** **Guide**

**Community Health Advocate (CHA) Program**

**Introduction**

1. What aspects of the CHA work well in your practice?
2. What CHA activities do you find most helpful?

**Integration into the practice**

1. Thinking about the initial integration of the CHA, what went well, what could be improved?
   1. Did you have enough support to integrate the CHA into your practice?
   2. Do you feel the CHA had adequate training?
2. How is the communication between you and the CHA?
   1. Do you hear from the CHA when the patient's needs have been met?
3. What aspects of the CHA program are challenging?

**Patient impact**

1. Does your office have the bandwidth to address social needs for your patients?
2. Do you have particular patient success stories with the CHAs that you could share?

**Conclusion**

1. Is there anything we have not asked you about today that you would like to share?

# Table 1: High-level data from the CHA program through December 2020 is outlined below. 17 CHAs have served over 1,900 patients and created 3,600 referrals. The top referral types vary by region, as each region has a diverse population with specific social needs. The program start dates and number of practices/CHAs vary by region, impacting total volume in some areas.

| Metric | Sacramento County | Monterey County | Butte County | Los Angeles County | All Regions |
| --- | --- | --- | --- | --- | --- |
| # of Social Needs Assessments | 2,327 | 426 | 67 | 18 | **2,838** |
| # of PATIENTS | ~1,000 | 843 | 42 | 21 | **1,906** |
| # of Referrals | 2,332 | 1,170 | 71 | 29 | **3,602** |
| Top Referral Type | Mental & Behavioral Health | Physical Health | Transportation | Food Assistance | **Physical Health** |

Notes: A patient may have more than one referral. The CHA program in Sacramento was the first to launch and has more CHAs than the other regions, which contributes to its higher volume of patients and referrals

# Table 2: The top referral types varied by practice type. Mental and Behavioral Health was most prevalent among both primary and specialty care practices.

| **Practice Type** | **Top Referral #1** | **Top Referral #2** | **Top Referral #3** |
| --- | --- | --- | --- |
| **Primary Care** | Mental & Behavioral Health | Physical Health | Benefits Navigation |
| **Specialty Care** | Mental & Behavioral Health | Housing & Shelter | Individual & Family Support |
| OB/GYN | Mental & Behavioral Health | Housing & Shelter | Clothing & Household Goods |
| Pulmonary Medicine | Mental & Behavioral Health | Benefits Navigation | Utilities |
| Orthopedic | Transportation | Food Assistance | Benefits Navigation |
| Endocrinology | Physical Health | Wellness | Transportation |

# Table 3: There were six key themes from provider, staff, and CHA interviews and focus groups on the perspectives of the CHA role, and what factors enabled his or her success.

| **Themes** | **Quotes from Practice Staff** |
| --- | --- |
| **CHAs with shared life experiences as patients** | *“I have experienced working with the patient and the patient is hesitant to tell me their social needs (female CHA talking about male population). Machismo is very established and I’m a 5’1 female, every time I have a new male patient referred by a male friend I’m surprised.” – CHA*  *“When you are looking for a health advocate, I think diversity is really important. And that really expands the outreach to different patients, depends on their ethnicity, their language. The CHA helped me a lot to reach out to patients where language was the biggest issue.” – Provider*  *“Our CHA has a social services background, and a military background. He has had much success connecting patients to Veteran services. He has experience with homeless populations, and he has had much success with housing, and food services.” – Practice staff* |
| **Establishing role clarity at the practice** | “*There was not a lot of information for all staff at the practices on the role of the CHA prior to implementation*” and “t*he first day I showed up and the clinic staff didn’t know what the position was.”– CHA*  *“For me, it felt nebulous because we weren’t totally sure how to use the CHA. What the actual role would be.” – Provider* |
| **Increased understanding of patient populations’ social needs** | “*This (CHA) is one of those things I didn’t really believe in. I was not an enthusiastic adopter. I was foolish. I thought people were doing better than they are, and I was wrong.” – Provider* |
| **Primary Care versus Specialty Care Practices** | *N/A* |
| **Improved patient engagement with their health and care** | *CHAs “give the patients a lifeline and a personal number they can call”. – CHA*  *“What [CHA] has done is go into the community and find the resources that are already there, but they are different bodies not really connected to each other. She went to a place where resources were available, she brought those resources into my clinic…the doctor is telling them to eat healthy, now this CHA is telling them where they can get these foods.” – Provider* |
| **Impact of COVID on social needs and CHA response** | *“Whatever routine the patient had before has mostly been put on hold. Causing tremendous stress.” – CHA*  *"We want to create a trusting relationship and because of the relationship (with the CHA) we’ve mitigated the need for some patients to be hospitalized." – Practice staff* |
